# A Novel Ketone-Supplemented Diet Improves Recognition Memory and Hippocampal Mitochondrial Efficiency in Healthy Adult Mice

**DOI:** 10.3390/metabo12111019

**Published:** 2022-10-25

**Authors:** Erin R. Saito, Cali E. Warren, Cameron M. Hanegan, John G. Larsen, Johannes D. du Randt, Mio Cannon, Jeremy Y. Saito, Rachel J. Campbell, Colin M. Kemberling, Gavin S. Miller, Jeffrey G. Edwards, Benjamin T. Bikman

**Affiliations:** Department of Cell Biology and Physiology, Brigham Young University, Provo, UT 84602, USA

**Keywords:** ketogenic diet, hippocampus, mitochondrial efficiency, recognition memory, metabolism

## Abstract

Mitochondrial dysfunction and cognitive impairment are common symptoms in many neurologic and psychiatric disorders, as well as nonpathological aging. Ketones have been suggested as therapeutic for their efficacy in epilepsy and other brain pathologies such as Alzheimer’s disease and major depressive disorder. However, their effects on cognitive function in healthy individuals is less established. Here, we explored the mitochondrial and performative outcomes of a novel eight-week ketone-supplemented ketogenic (KETO) diet in healthy adult male and female mice. In a novel object recognition test, KETO mice spent more time with the novel, compared to familiar, object, indicating an improvement in recognition memory. High-resolution respirometry on permeabilized hippocampal tissue returned significant reductions in mitochondrial O_2_ consumption. No changes in ATP production were observed, yielding a significantly higher ATP:O_2_ ratio, a measure of mitochondrial efficiency. Together, these findings demonstrate the KETO diet improves hippocampal mitochondrial efficiency. They add to a growing body of evidence that suggests ketones and ketogenic diets are neuroprotective and metabolically and cognitively relevant, even in healthy adults. They also suggest that ketogenic lifestyle changes may be effective strategies for protecting against cognitive decline associated with aging and disease.

## 1. Introduction

Mitochondria wear a number of different hats within the cell. Although they are also central to processes of Ca^2+^ homeostasis, apoptosis, and reactive oxygen species production, their most critical role, arguably, lies in ATP production and cellular nutrient metabolism. In the brain, neurons are particularly dependent on mitochondria-produced energy for the maintenance of membrane potential, neurotransmission, and synaptic plasticity [1], all of which are essential to cognitive health. Mitochondrial dysfunction and dysregulated energy metabolism, two of the hallmarks of brain aging [2], have been suggested to drive aging and disease-induced cognitive decline. Therefore, identifying ways to improve mitochondrial health, pharmacologically and behaviorally (e.g., diet, exercise, etc.), represents a promising strategy to prolong cognitive health and protect against dementias such as Alzheimer’s disease (AD), as have been previously suggested [3,4,5].

The brain only makes up 2–3% of the human body mass, yet receives roughly 15% of the body’s cardiac output [6,7] and accounts for approximately 20% of its fuel and oxygen consumption [8]. As a metabolic giant of an organ, the brain is highly sensitive to even subtle changes in energy homeostasis. The hippocampus, a structure within the medial temporal lobe, functions as the brain’s primary memory center and is essential for learning and other cognitive functions. In neurons within the hippocampus, mitochondria are essential for fueling energetically expensive processes of synaptic plasticity, which constitute the molecular mechanism of learning and memory formation [9,10]. In mice, alleviating synaptic mitochondrial dysfunction in the hippocampus is sufficient to decrease age-associated cognitive impairment [11].

The brain is a “picky eater” and is limited to two main fuel sources—glucose and ketones. Under low glucose conditions, such as fasting, exercising, and low-carbohydrate ketogenic diets (KD), the liver produces ketones—most prevalently, acetoacetate (AA) and beta-hydroxybutyrate (BHB)—from fatty acids to fuel the body. Ketones are metabolized by the brain even when adequate glucose is available [12], sparing glucose proportionally to plasma ketone concentrations [13,14], and can account for almost 70% of the brain’s energy requirements [15].

Low-carbohydrate, high-fat dietary regimens that mimic aspects of fasting via ketogenesis, are widely accepted as neuroprotective due to their well-established use in treating refractory and childhood epilepsy [16,17]. Since their initial implementation over 100 years ago [16], ketogenic diets have regained attention in recent decades due to their expanding utility in the treatment and management of other brain disorders involving bioenergetic impairments [18]. These include Alzheimer’s [19,20,21] and Parkinson’s disease [22,23], cancers [24,25], and mental health disorders such as major depressive disorder [26,27] and schizophrenia [28,29]. In these conditions, ketogenic diets have been shown to reduce inflammation and oxidative stress, increase the activity of neurotrophic factors, and enhance energy metabolism [30,31,32].

Ketone esters (KE), used to mimic the KD, are effective at inducing rapid, sustained elevations in circulating ketones. The oral administration of KEs has also displayed clinical relevance in multiple neurological disease states in both rodents and humans. Once ingested, KEs are hydrolyzed by nonspecific gastric and tissue esterases. This frees ketones from their backbone molecule, often butanediol [33], which is also ketogenic and is oxidized in the liver to BHB via alcohol and aldehyde dehydrogenase [34]. Ketones are then oxidized by metabolically active tissues to produce ATP. The KE used in this study, R,S-1,3-butanediol acetoacetate diester, is a nonionized sodium-free precursor of ketone bodies AA and BHB that has been previously demonstrated as safe and effective in elevating blood ketone concentrations [33].

The utilization of ketones and ketogenic diets in the treatment of neurological disease states and mental health disorders is well-founded. Although it has been demonstrated that a cyclic KD extends the health span and cognitive function in aging mice [35], its utility in protecting against cognitive aging in healthy adults is less established. In the current study, we explore the effects of a novel eight-week KE-supplemented ketogenic (KETO) diet. We test the hypothesis that the KETO diet improves behavioral recognition memory and hippocampal mitochondrial bioenergetics in healthy adult, wildtype mice. The results presented here suggest that ketogenic intervention may represent an effective strategy for improving hippocampal metabolism and cognition in healthy adults.

## 2. Materials and Methods

### 2.1. Animals and Diet

Studies were conducted in accordance with the principles and procedures outlined in the National Institutes of Health Guide for the Care and Use of Laboratory Animals and were approved by the IACUC (Institutional Animal Care and Use Committee) at Brigham Young University. Additionally, experiments have been reported in compliance with the ARRIVE guidelines 2.0 on reporting animal experiments.

Adult (average age of 10.7 months at sacrifice, SEM = 0.88) male and female C57BL/6 mice were purchased from Jackson Laboratories, group-housed, and maintained at 22 ± 1 °C, 60–70% humidity, and a 12-h light–dark cycle. Mice were randomly divided into two groups and given ad libitum access to food—a control rodent diet (CON) or a ketogenic diet supplemented with an exogenous ketone ester (KETO)—and water. The CON diet was purchased from LabDiet (5001). The KETO diet was made up of a lard-based KD paste (BioServ (F3666), R,S-1,3-butanediol acetoacetate diester (ketone ester, KE; Disruptive Enterprises), and sugar-free peanut butter (Jif) to improve palatability and prevent excessive weight loss.

The KETO diet was mixed freshly twice a week or more often as was necessary and, by weight, was composed of 90% KD, 5% KE, and 5% peanut butter, which translated to a macronutrient composition of 90% kcal fat, 4.7% kcal protein, 2% kcal carbohydrate, and 3.3% kcal KE (Table 1). This assumed a caloric density of 4.7 kcal/g for the KE, previously established in other work [36]. Weight was measured once a week, or more frequently if body weight appeared to be declining, especially in KETO mice during the first two weeks of diet conditioning. To maintain as many mice as possible in the study, if the weights approached the 20% cutoff, the concentration of peanut butter was increased (and keto diet paste reduced) to promote palatability and prevent further weight loss. Mice were excluded if >20% of their initial body weight was lost.

Ketone and glucose measurements were taken once a week to ensure diet efficacy. Blood was drawn via tail tip amputation and tail massage, which has been suggested as a superior method for reducing animal distress compared to tail vein incision and facial vein puncture [37]. Precision Xtra blood glucose and ketone meters (Abbott) were used to measure the blood glucose and R-BHB concentrations. BHB is a chiral molecule, with R and S enantiomers, which are differentially metabolized [38]. R-BHB is the primary product of normal metabolism and is readily metabolized to acetyl CoA to produce ATP. S-BHB, however, is not a normal product of rodent nor human metabolism. Although it is largely converted to R-BHB, the metabolism of S-BHB is much slower, and can account for sustained elevations in ketones with KE administration [39]. Although we did not measure the blood S-BHB, the racemic R,S-1,3-butanediol acetoacetate diester likely elevated both R- and S-BHB.

Researchers collecting data were not blinded to the animals’ diets.

### 2.2. Two-Object Novel Object Recognition

The novel object recognition (NOR) assay is a simple, widely used behavioral test of memory that does not rely on food reward (radial arm maze) or survival instincts (Morris water maze) but on rodents’ innate predisposition to explore novelty in the absence of external stimuli. It is a widely used test of nonspatial memory that can be configured to probe multiple aspects of memory [40] without inducing significant stress. Here, we used the basic two-object procedure due to its more common use in the recent literature [41,42,43].

Preliminary pilot tests were conducted with a small group of mice (3–6) to confirm that mice spent at least 20 s with each object within the allotted 8 min and that there were no initial biases toward either object (discrimination index of approximately 0).

The two-object NOR was carried out in a round, plastic, open field arena with a diameter of 45.72 cm (18 in) and height of 60.96 cm (24 in) and recorded with a GoPro for later analysis with AnyMAZE software. The protocol consisted of one day of habituation (T0), one day of training (T1), and one day of testing (T2), with 24 h between each session. During habituation, mice were placed in the middle of the open arena and left to explore for 8 min. Once finished, mice were placed in a separate holding cage until their home cage was empty; at which point, they were returned. The arena was thoroughly cleaned between each mouse. On T1, two identical objects were placed in the arena, equidistant from the opposite wall. Mice were placed in the arena facing the opposite wall, equidistant from both objects to prevent directional biases upon release. Mice were left to navigate and interact with the identical objects for 8 min. Similar to habituation, mice were placed in a separate holding cage after the trial until their home cage was empty. On T2, one of the familiar objects (FO) was replaced with a novel object (NO). Again, mice were placed in the arena facing the opposite wall and left to explore the objects for 8 min.

Using AnyMAZE software, the amount of time mice spent with each object, defined as the time spent touching the object or within 2 cm facing the object, velocity, and distance traveled were measured. A discrimination index ((NO − FO)/(NO + FO) × 100) was calculated. Mice that did not reach a 20-s minimum of exploration for both objects in T1 within 8 min were excluded from the analysis.

### 2.3. Brain Slice Preparation

The methods used to prepare brain slices were similar to those described previously [44,45,46]. Mice were anesthetized with isoflurane in a vapomatic chamber and subsequently decapitated via guillotine. Following decapitation, the brains were rapidly extracted and placed in ice-cold, oxygenated artificial cerebrospinal fluid (ACSF) containing 119 mM NaCl, 26 mM NaHCO_3_, 2.5 mM KCl, 1 mM NaH_2_PO_4_, 2.5 mM CaCl_2_, 1.3 mM MgSO_4_, and 10 mM glucose saturated with 95% O_2_ and 5% CO_2_, pH 7.4). Salts were purchased from Sigma-Aldrich (St. Louis, MO, USA), Mallinckrodt-Baker (Phillipsburg, NJ, USA), or Fisher Scientific (Waltham, MA, USA) and dissolved in double-distilled water (ddH_2_O). A vibratome (Leica) was used to cut coronal hippocampal slices (350 μm) in ice-cold, oxygenated ACSF. The hippocampi were then dissected out and transferred to a holding chamber containing room temperature oxygenated ACSF. Here, the hippocampi were either set aside for mitochondrial respirometry or snap-frozen in liquid nitrogen and stored at −80 °C for ATP quantification, Western blots, and other assays.

### 2.4. Mitochondrial Respirometry

Mitochondrial oxygen consumption rates were determined at 37 °C from freshly isolated hippocampal tissue using the Oroboros O2K Oxygraph (Innsbruck, AUT) with MiR05 respiration buffer, as described previously [47]. Prior to loading in the O2K machine, the hippocampi were permeabilized in 0.05 mg/mL of saponin (Sigma-Aldrich) in MiR05 for 30 min at 4 °C. After the addition of hippocampi, the chambers were hyperoxygenated to ∼350 nmol/mL and shut. A stable baseline respiration rate was established, which took approximately 5 min. Changes in the respiration rates were then determined following a substrate–uncoupler–inhibitor–titration protocol. The electron flow through complex I was supported by glutamate  +  malate (GM, 10 and 2 mM, respectively) to determine the leak oxygen consumption (O_GM_). Following stabilization, ADP (2.5 Mm) was added to determine the oxygen consumption associated with oxidative phosphorylation (O_ADP_). Succinate (S) was then added to support complex I and II electron flow into the Q-junction (O_S_). To maximize the electron transport capacity given these substrates, the chemical uncoupler carbonyl cyanide 4-(trifluoromethoxy) phenylhydrazone (FCCP) was added (0.05 Μm, O_FCCP_). The respiratory control ratio (RCR) and CII factor were determined by calculating the ratio of O_ADP_:O_GM_ and the difference between O_S_ and O_ADP_, respectively.

Samples were then collected and stored at −20 °C. Protein concentrations were measured via the BCA assay (Perkin Elmer, Waltham, MA, USA), and the respiration rates were normalized to the protein concentration.

### 2.5. ATP Quantification

The ATP concentrations were quantified from hippocampal homogenates using an ATPLite Luminescence Assay kit (Perkin Elmer). Frozen hippocampi were thawed on ice and homogenized via sonication in approximately three volumes of ice-cold ATP-stabilizing buffer (PBS containing 20 mM glycine, 50 mM MgSO_4_, and 4 mM EDTA), similar to methods described previously [48]. Homogenates were diluted in ddH_2_O and were transferred to opaque, white, 96-well plates in volumes of 100 µL per well. The ATPLite protocol was then followed. ATPLite lysis buffer was added (50 µL) to each well, and the plates were agitated for 5 min at 700 rpm at room temperature. ATPLite substrate solution was then added (50 µL) to each well. The plates were covered with aluminum foil, agitated for an additional 5 min at 700 rpm at room temperature, and dark-adapted for 10 min. Luminescence was subsequently measured with a Victor Nivo Multimode Plate Reader (Perkin Elmer), and the ATP concentration was normalized to the protein concentration via the BCA assay (Perkin Elmer).

### 2.6. Western Blot

Frozen hippocampi were thawed on ice and sonicated with RIPA buffer supplemented with protease and phosphatase inhibitors at a final concentration of 1% *v*/*v* each (P8340, P0044, Sigma-Aldrich). Protein concentrations were determined via the BCA protein assay kit (Pierce), and the sample volumes were adjusted to load 20 µg of protein per lane. Sample buffer (4× Laemmli buffer, 10% beta mercaptoethanol) was added to each sample and heated at 95 °C for 10 min. Samples were resolved by SDS/PAGE (12%) at 70 V and transferred onto nitrocellulose membranes. The membranes were blocked using 5% nonfat dry milk dissolved in 1× tris-buffered saline with 0.1% tween (TBST) for 30 min and incubated with primary antibodies diluted to their final concentrations in 5% BSA, 0.02% sodium azide (see Table 2 for primary antibody information and dilutions) overnight at 4 °C. Membranes were then washed with TBST and incubated with fluorescently labeled donkey anti-mouse and anti-rabbit secondary antibodies (LI-COR) at a 1:2500 dilution at room temperature for one hour. Membranes were washed with TBST and imaged on a LI-COR Odyssey CLx. Target protein fluorescence was normalized to the loading control (β-actin) fluorescence. Target protein: β-actin ratios were compared between the CON and KETO groups.

### 2.7. Statistics

Data are presented as the means ±SEM. Differences between the CON and KETO means were compared using Student’s *t*-tests (GraphPad Prism; Microsoft Excel). The Shapiro–Wilk test was used in conjunction with the visual assessment of q–q norm plots to determine normality for all analyses. The significance was determined at *p* < 0.05.

### 2.8. Sex as a Biological Variable

The rodent estrous cycle lasts approximately 4–5 days, in which time 17β-estradiol levels gradually increase until proestrus, when 17β-estradiol quickly rises and falls. Circulating estradiol in the mouse is highest during the proestrus phase of the estrus cycle and has been shown to alter hippocampal physiology and cognition [49,50]. High estrogen during the proestrus phase of the cycle has been shown to enhance LTP [51], BDNF expression [52], and reduce ROS production [53]. To control for fluctuations in circulating estrogen, female estrus cycles were visually tracked. The vaginal opening was evaluated based on the criteria described by Champlin et al. [54]. Mice underwent behavioral testing and sacrifice when mice were not in proestrus.

## 3. Results

### 3.1. Ketogenic Diet Elevates Blood Beta-Hydroxybutyrate

**Figure 1:**
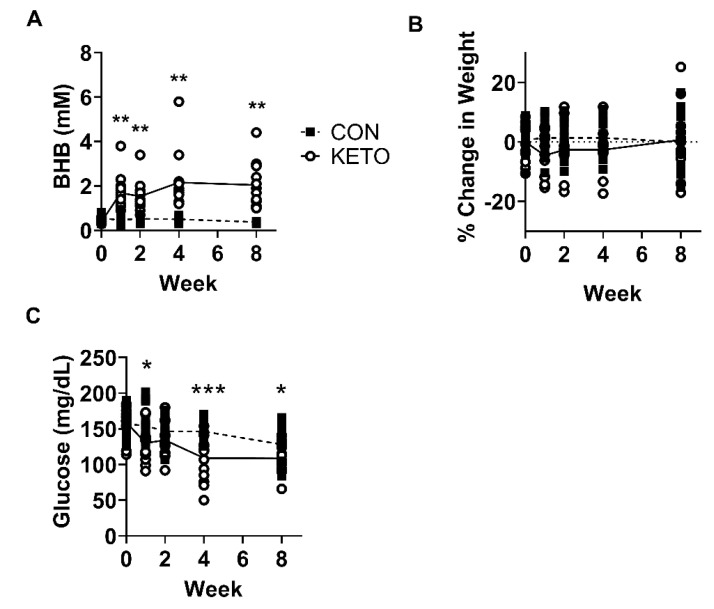
Ketogenic diet elevates blood ketone β-hydroxybutyrate (BHB). (**A**) Blood BHB (CON, *n* = 15; KETO, *n* = 14), (**B**) weight (CON, *n* = 15; KETO, *n* = 11), and (**C**) blood glucose (CON, *n* = 12; KETO, *n* = 13) over the course of an 8-week ketogenic diet in C57BL/6 mice. * *p* < 0.05, ** *p* < 0.01, and *** *p* < 0.001.

In the initial tests, male and female C57BL/6 mice placed on the ketogenic lard diet (BioServ F3666) alone displayed only mild elevations in blood ketone concentrations (data not shown). Supplementing the KD with 5% exogenous ketone ester (R,S-1,3-butanediol acetoacetate diester) displayed significantly higher levels (Figure 1A; week 0 *p* = 0.01, week 1 *p* < 0.001), week 2 *p* < 0.001, week 4 *p* < 0.001, and week 8 *p* < 0.001) of blood BHB and significantly lower levels of blood glucose (Figure 1B; week 0 *p* = 0.42, week 1 *p* = 0.016, week 2 *p* = 0.16, week 4 *p* = 0.001, and week 8 *p* = 0.015) compared to CON mice throughout diet conditioning. Despite these data, the weights were not significantly different between CON and KETO mice (Figure 1C; week 0 *p* = 0.56, week 1 *p* = 0.16, week 2 *p* = 0.53, week 4 *p* = 0.48, and week 8 *p* = 0.54).

### 3.2. Ketogenic Diet Improves Recognition Memory and Locomotion

**Figure 2:**
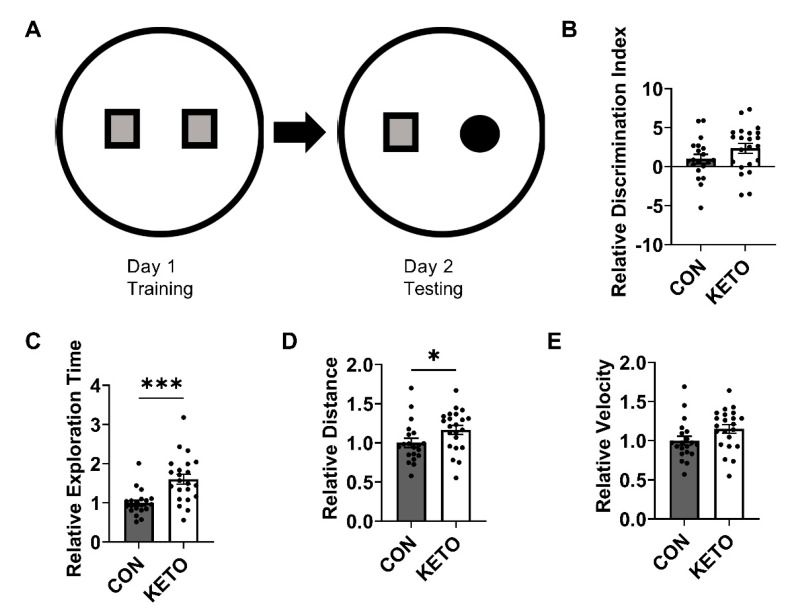
Ketogenic diet enhances novel object recognition and locomotion. (**A**) Two-object novel object recognition test layout and resulting measures of recognition memory ((**B**) discrimination index and (**C**) exploration time) and locomotion ((**D**) distance and (**E**) velocity). CON *n* = 20; KETO *n* = 22. * *p* < 0.05, *** *p* < 0.001.

At the end of diet conditioning, CON and KETO recognition memory was assessed in the novel object recognition test. During NOR testing (T2), KETO mice spent significantly more time (Figure 2C; *p* < 0.001) with the novel compared to a familiar object. However, the discrimination index, a measure of the mouse’s ability to discriminate the novel from familiar object, indicated a trend toward significance (Figure 2B; *p* = 0.12). Measures of the average distance traveled and velocity indicated general increases in locomotion with the KETO treatment, such that KETO mice traveled significantly further (Figure 2D; *p* = 0.047) and strongly trended toward a significant increase in velocity (Figure 2E; *p* = 0.066) compared to CON mice.

### 3.3. Ketogenic Diet Enhances Hippocampal Mitochondrial Efficiency

**Figure 3:**
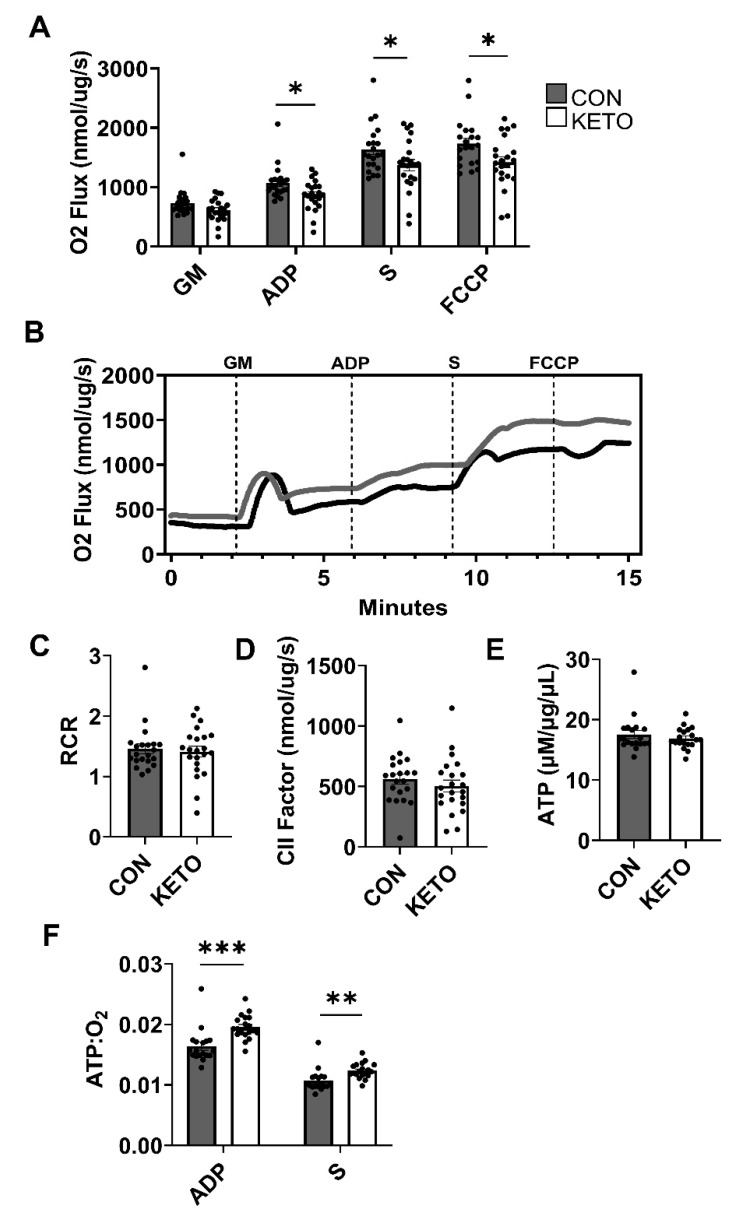
Ketogenic diet improves hippocampal mitochondrial efficiency. (**A**) Rates of mitochondrial oxygen consumption were measured via a substrate–uncoupler–inhibitor–titration protocol from hippocampi following an 8-week control or ketogenic diet. The protocol included the addition of glutamate and malate (GM), ADP, succinate (S), and carbonyl cyanide 4-(trifluoromethoxy) phenylhydrazone (FCCP). (**B**) Representative traces of oxygen consumption are plotted versus time with indicated substrate additions. (**C**) Respiratory control ratios (RCR) and (**D**) complex II-associated respiratory flux (CII factor) were calculated (CON, *n* = 21; KETO, *n* = 22) (**E**) ATP concentration and (**F**) ATP:O2 flux ratios were quantified (CON, *n* = 18; KETO, *n* = 19).* *p* < 0.05, ** *p* < 0.01, and *** *p* < 0.001.

High-resolution respirometry (Figure 3A,B) indicated that the KETO diet significantly reduced the mitochondrial respiration rate of hippocampi with the addition of ADP (O_ADP_, *p* = 0.013). ADP sustains oxidative phosphorylation supported by the complex I-mediated electron flow. The KETO diet also significantly reduced the mitochondrial oxygen consumption rate with the addition of succinate (O_S_, *p* = 0.044), which supports the complex II-mediated electron flow, and carbonyl cyanide-4-(trifluoromethoxy)phenylhydrazone (O_FCCP_, *p* = 0.021), which induces the maximum oxygen flux due to uncoupling. A strong trend toward significance was observed with the addition of glutamate and malate (O_GM_, *p* = 0.053), which support complex I-mediated respiration associated with the proton leak. Representative traces of oxygen consumption with the addition of GM, ADP, S, and FCCP were plotted versus time in Figure 3B. The time at which O_2_ flux began to plateau was designated as time = 0. The plateau occurred within 5 min following the addition of hippocampi to the respiration chambers and directly prior to starting the SUIT protocol.

ATP quantification indicated no significant change in ATP concentration between the CON and KETO hippocampi (Figure 3E; *p* = 0.42). However, the ratio of ATP to oxygen consumed with the activation of oxidative phosphorylation (ATP:O_ADP_) demonstrated improvements in mitochondrial coupling and efficiency (Figure 3F; *p* < 0.001). Additionally, ATP:O_S_ demonstrated a significant improvement in mitochondrial efficiency (Figure 3F; *p* = 0.005). The KETO ATP:O_ADP_ ratios were, on average, almost 20% higher (19.7%) than the CON ratios. On average, the KETO ATP:O_S_ ratios were almost 12% higher than the CON ratios. The respiratory control ratio (RCR), a general measure of the general mitochondrial fitness (Figure 3C), and the complex II factor (CIIF), a measure of complex II’s contribution to the respiration rate (Figure 3D), indicated no significant differences between the CON and KETO mice (*p* = 0.7, *p* =0.39).

### 3.4. Ketogenic Diet Induces Sex-Specific Hippocampal Mitochondrial Complex V Expression

**Figure 4:**
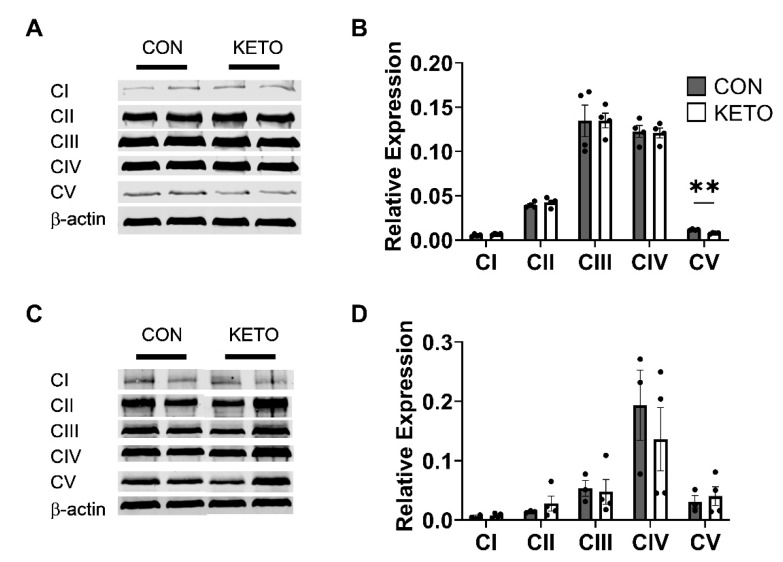
Ketogenic diet significantly reduces complex V expression in female mice. Representative Western blots against mitochondrial complexes I–V in (**A**,**B**) female (CON, *n* = 4; KETO, *n* = 4) and (**C**,**D**) male (CON, *n* = 3; KETO, *n* = 4) mice and their relative expression. ** *p* < 0.01.

To determine whether changes in the mitochondrial respiration rates were due to changes in the expression of the mitochondrial complexes, Western blots were performed probing for complexes I–V. Together, male and female mice did not display any significant changes in the expression of the mitochondrial complexes (CI *p* = 0.28, CII *p* = 0.33, CIII *p* = 0.76, CIV *p* = 0.52, and CV *p* = 0.71). However, when compared separately, female mice on the ketogenic diet displayed a significant reduction in the expression of CV (Figure 4A,B; *p* = 0.001), while male mice did not (Figure 4C,D; *p* = 0.64).

### 3.5. Ketogenic Diet Does Not Alter the Expression of Hippocampal Mitochondrial Dynamics Proteins

**Figure 5:**
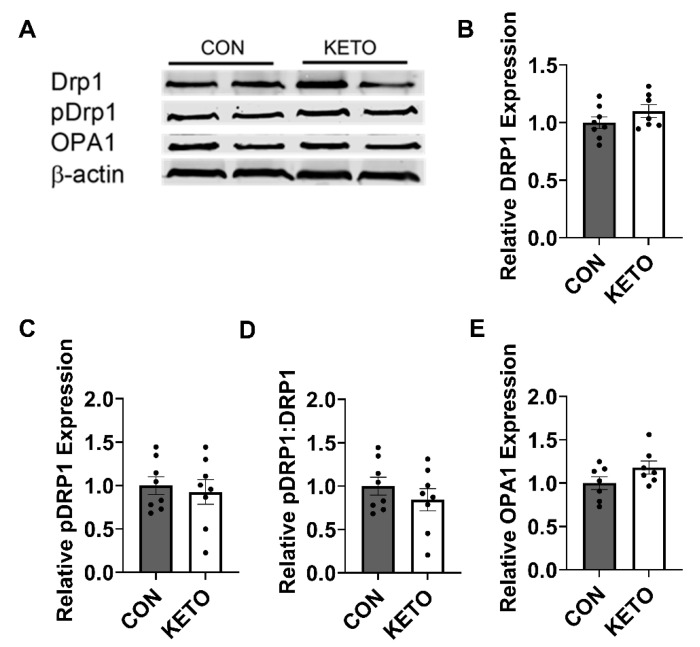
Ketogenic diet does not alter the hippocampal mitochondrial dynamics. (**A**) Representative Western blots against DRP1 (CON, *n* = 8; KETO, *n* = 7), pDRP1 (CON, *n* = 8; KETO, *n* = 8), OPA1 (CON, *n* = 7; KETO, *n* = 7), and their (**B**–**E**) respective relative quantifications.

To determine whether a ketogenic diet alters the expression of proteins involved in mitochondrial dynamics, Western blots were performed probing for DRP1, a mitochondrial fission protein, and OPA1, a mitochondrial fusion protein (Figure 5A). DRP1 expression was not significantly altered by KETO conditioning (Figure 5B; *p* = 0.21). pDRP1 expression, expressed as pDRP1:DRP1, was similarly unaffected (Figure 5C; *p* = 0.36). OPA1 expression, however, exhibited a trend toward significance (Figure 5E; *p* = 0.11).

## 4. Discussion

The current study provides further evidence of metabolically relevant, neuroprotective effects of ketones within the brain, adding to a large body of evidence that suggests manipulating peripheral nutrient metabolism through diet can offer neuroprotection. Our findings are consistent with previous studies utilizing KE-based diets that demonstrate improvements in behavioral anxiety and memory [55,56], as well as hippocampal metabolism [57]. Here, we assessed the effects of a novel ketone-supplemented KETO diet on recognition memory and hippocampal bioenergetics in healthy adult mice and found that the diet improves cognitive function and mitochondrial efficiency.

At the inception of this study, C57BL/6 mice were initially placed on a control rodent chow (CON, LabDiet 5001) or a lard-based KD alone (KD, Bioserv F3666). The CON-fed mice displayed elevated blood BHB levels similar to the KD-fed mice (Figure 1A; week 1, 0.47 mM). This was surprising, as we found little evidence of this in previously published reports on the topic. Although the KD significantly elevated the blood BHB levels compared to the CON, the difference in blood concentration was minimal (~0.2 mM, data not shown) and, we deemed, not robust enough to justify using the KD alone to quantify the effects of ketones on hippocampal physiology. The KD lard-based diet was then supplemented with the ketone ester, R,S-1,3-butanediol acetoacetate diester, which further elevated the blood BHB (Figure 1A; week 1, 1.69 mM), allowing us to proceed with the study. These findings highlight the necessity of directly measuring blood BHB levels in rodent studies exploring the effects of ketones on physiology in the context of ketogenic diets. These findings also call attention to the innate differences in nutrient metabolism between humans and mice, such that mice have a basal metabolic rate per kg of body mass that is approximately seven time higher than humans [58] and, as we observed, have a greater predisposition to a ketogenic state without severe carbohydrate restriction (CON 57.9% carbohydrate, 13.4% fat, and 28.7% protein). These must be taken into consideration when evaluating the translational potential of ketogenic diet research in mice and other model organisms.

We observed KETO-induced improvements in behavioral recognition memory (Figure 2) that were accompanied by enhancements in the hippocampal mitochondrial efficiency (Figure 3). High-resolution respirometry revealed that, with the addition of glutamate, malate, ADP, and succinate, which induce CI and CII electron flow into the Q junction, the KETO diet significantly reduced the oxygen flux (Figure 3A) but maintained the same concentration of ATP as the CON diet (Figure 3D). In other words, the KETO diet significantly improved mitochondrial efficiency within the hippocampus or ATP produced per unit of oxygen consumed (Figure 3E). We also observed a significant reduction in oxygen consumption with glutamate, malate, and ADP addition or complex I (NADH:ubiquinone oxidoreductase) contribution to oxidative phosphorylation (Figure 3E). Paired with the unchanged ATP concentrations, this also produced a significantly higher rate of ATP:O2, which suggests that both complex I and II (succinate–coenzyme Q reductase) are likely involved in the KETO-induced improvements in mitochondrial efficiency. Overall, these data indicate that hippocampal mitochondrial respiration is more tightly coupled to ATP production under prolonged KETO compared to CON exposure.

This oxygen-sparing effect has implications for the conditions of cerebral hypoxia, for example, traumatic brain injury (TBI), stroke, and other forms of ischemia in which the blood oxygen supply to the brain is occluded. The hippocampus has been demonstrated as particularly vulnerable to hypoxic stress and is one of the first brain regions to display degeneration [59,60]. Rats placed on a KD after a controlled cortical impact injury have demonstrated a decrease in cortical contusion volume and a reduction in cortical and hippocampal neurodegeneration [61]. Other studies have explored the effect of ketogenic diets on TBI and have demonstrated that the diet has protective effects against apoptosis and delays TBI-induced deficits in energy metabolism in juvenile rats [62,63]. The results presented here suggest a potential additional mechanism: KETO-induced oxygen sparing may promote cell survival by extending oxygen supply. This is especially impactful, because clinical trials assessing the safety and efficacy of the KD as a treatment for TBI are ongoing (NCT03982602).

Mitochondria are incompletely coupled, meaning during oxidative phosphorylation, some of the redox energy is dissipated rather than coupled to ATP production. This occurs as a regulated process via uncoupling protons but also occurs as an unregulated background proton leak across the inner mitochondrial membrane (IMM). Proton leak-associated oxygen consumption exhibited a very strong trend (*p* = 0.053) toward significant reduction in the KETO hippocampus compared to the CON. Glutamate and malate feed into the malate–aspartate shuttle, which transports NADH-reducing equivalents from the cytoplasm to the mitochondrial matrix, where they have access to mitochondrial complex I. In the absence of ADP, this activates a non-phosphorylated respiratory state in which oxygen consumption is associated with a proton leak across the IMM back into the matrix [64]. Although not the only means of uncoupling, a proton leak is the primary mechanism that uncouples mitochondrial oxygen consumption from ATP production [65]. Increased proton conductance (leak) across the IMM has been theorized to minimize oxidative damage at the expense of compromising energy homeostasis [66]. However, these data suggest that the KETO diet decrease the hippocampal proton leak without compromising energy production. While we did not quantify the expression of uncoupling proteins, the strong trend toward reduction in proton leak-associated oxygen consumption with the KETO treatment indicated less leak-associated uncoupling. This is consistent with our ATP data, in which we observed a significant increase in the ATP:O2 ratio and tighter bioenergetic coupling (Figure 3E).

Proton permeability of the IMM is susceptible to modulation, which can result in fluctuations in uncoupling. This includes physical changes in the lipid composition and structure of the IMM lipid bilayer and the presence or absence of uncoupling proteins [67,68]. For example, hyperthyroidism in rats has been shown to increase liver IMM proton permeability. This occurs via thyroid hormone-induced changes in the phospholipid bilayer components that increased the IMM surface area [69]. Environmental factors such as temperature and diet have also been shown to alter the IMM lipid composition in rodents and other organisms and, consequently, ATP:O2 ratios and animal performance [70]. Therefore, although we did not assess this directly, it is possible that the KETO diet we used in this study altered the hippocampal IMM lipid composition to decrease the proton leak and improve the mitochondrial efficiency.

Oxidative damage is one of the hallmarks of brain aging [2] and is a well-established driver of cognitive decline [71,72] and neurodegenerative disease [73,74]. Mitochondria are a significant source of ROS. This is due to their role in oxidative phosphorylation in which molecular oxygen is reduced to water by complex IV (cytochrome C oxidase) of the electron transport system. Within the mitochondria, complexes I and III (ubiquinol–cytochrome c reductase) are the primary sites of ROS production [75]. Although we did not directly quantify the ROS, it is possible the KETO treatment also decreased the ROS production due to a reduced mitochondrial O_2_ flux and improved the energy efficiency. This would be consistent with the literature demonstrating ketones reduce oxidative stress [76,77,78] but would require further testing.

In this study, the novel object recognition test was selected as an indicator of cognitive function. The hippocampal formation is organized in a way to support memory storage and recall. Following exposure to a stimulus (for example, the visual stimulus of an object), the hippocampus must determine whether the information is novel and requires encoding as a new memory or old and requires retrieval of a previously encoded memory. These rely on hippocampus-dependent processes of pattern completion and separation. Pattern completion, the ability to retrieve whole memories (neural representations) from partial or noisy cues, is dependent on CA3 recurrent collaterals, while pattern separation, the process of distinguishing similar experiences by minimizing overlap in neural representations, is dependent on mossy fiber inputs to CA3 [79]. Together, the significant increase in time with the NO combined with the trend toward significance in the increased discrimination index (Figure 2B,C) indicated KETO-induced improvement in recognition memory, which requires both pattern separation and completion. Although we did not distinguish changes in the bioenergetics in dentate gyrus granule cells from CA3 pyramidal neurons, here, we display general enhancements in the hippocampal bioenergetic efficiency that correlate with improvements in the recognition memory (Figure 2 and Figure 3).

More generally, the ability to recognize stimuli that have been previously experienced relies on recollection of the stimulus in the context of its original exposure and general familiarity with aspects of the stimulus. Historically, recollection and familiarity have been thought to be anatomically distinct processes, whereby recollection is mediated by the hippocampus and familiarity is mediated by the surrounding perirhinal cortex [80,81]. However, more recent evidence suggests the hippocampus is necessary for both [82,83,84]. Alzheimer’s disease (AD) is the most common form of senescence-related dementia, affecting approximately 6.5 million individuals in the United States and approximately 55 million globally. AD is characterized by neurodegeneration that progressively impairs cognition and behavior. Research suggests that both recollection and familiarity are affected in AD patients—recollection at all stages and familiarity at the later stages of disease progression [85,86]. Although we did not assess the effects of the KETO diet on recognition memory in a mouse model of AD, the results presented here suggest that ketones may be therapeutic due to their ability to improve recognition via hippocampal mitochondrial function.

In the NOR, we also observed a significant increase in distance traveled and a trend toward a significant increase in the relative velocity (Figure 2D,E) that indicated KETO treatment also enhanced the locomotion. Combined, these cognitive and locomotive data agree with previous research demonstrating a KD enhances the physical and cognitive performance in rats [87].

A recent RNA-seq study showed that a 90-day KD induced changes in the metabolic gene expression in neurons isolated from whole brain homogenates [88]. Researchers found that the KD increased the expression of oxidative phosphorylation-related genes in neurons but not astrocytes. They speculated that the upregulated expression of mitochondrial complex genes would likely cause an increase in oxygen consumption to enhance oxidative phosphorylation. In our study, we observed the opposite—a reduction in mitochondrial oxygen consumption—with the stimulation of mitochondrial complexes I–V (Figure 3A), and no change in the expression of the complexes themselves, with the exception of complex V in the female mice (Figure 4). We determined this difference in female mice may be a female-specific effect independent of circulating 17β-estradiol, as we accounted for estrogen via visually tracking the estrus cycle.

While it could be easy to label the results presented here as conflicting, there are several differences that must be accounted for. The current study assessed hippocampus-specific physiology, while the RNA-seq study analyzed whole brain homogenates and isolated neurons and astrocytes. Since we used hippocampal homogenates, it is possible that the expression of neuronal mitochondrial complexes was, in fact, increased with KETO treatment. However, our data is limited to hippocampal homogenates, making it impossible to distinguish neuronal versus astrocytic gene expression.

More generally, the brain is a heterogenous organ, containing substantial populations of glial cells that support neuronal activity. In addition to their functions in the blood–brain barrier permeability, myelin production, and immune function, glial cells—notably, astrocytes and oligodendrocytes—play central roles in brain energy metabolism. They provide aerobic glycolysis-derived lactate to neurons, especially during periods of high synaptic activity [89]. Although it was historically believed that glia occurred in the brain at a high 10:1 glia:neuron ratio, more recent evidence suggests a global ratio closer to 1:1 [90], which also varies by brain region [91]. As a substantial proportion of cells are in the brain, it is worthwhile to note that we did not assess the contributions of specific glial populations to the results presented here. While they likely contributed to the significant changes we observed in mitochondrial efficiency and behavior, further study will be required to elucidate their specific contributions.

Mitochondria are remarkably dynamic organelles that vary in size, number, and location to meet the local energetic demands. Variations in the mitochondria size and number are mediated by processes of fission and fusion. The balance of fission and fusion have effects on mitochondrial bioenergetics, such that mitochondrial fusion is generally associated with an increase in mitochondrial efficiency and fission with a decrease in efficiency [92], and is, therefore, tightly regulated. Due to this, we explored the effects of the KETO diet on the expression of DRP1, a protein involved in mitochondrial fission, and OPA1, a protein involved in fusion. Here, we demonstrate no significant change in DRP1 or OPA1 expression, despite a significant increase in mitochondrial efficiency with KETO treatment. While we did not visualize the mitochondria directly, together, these suggest that the improvement in energy efficiency within the hippocampus occurred independent of changes in the mitochondrial dynamics.

Similar to other research investigating the effects of ketogenic diets on physiology, it is difficult to parse out whether the observed effects were due to carbohydrate restriction, the direct effects of the ketones themselves, or both. Additionally, because the caloric intake was not directly quantified and compared between CON and KETO mice, it is possible that some effects—for example, the significant reduction in blood glucose—were due to reduced food consumption rather than the direct effects of the KETO diet. However, the lack of significant changes in body weight throughout the KETO treatment argued against this. In total, these data confirmed that environmental factors, such as diet, can induce variations in mitochondrial efficiency [70] and that changes in efficiency can alter animal performances.

While age-related cognitive decline is a normal symptom of nonpathological aging, the dramatic rise in obesity over the last 50 years has proven to not only increase the risk of developing diseases with clear metabolic underpinnings such as insulin resistance, type 2 diabetes, and cardiovascular disease but also exacerbate cognitive aging [93,94]. In recent decades, it has become clearer that peripheral metabolic dysfunction has serious implications for brain health and cognitive function, as insulin resistance and its comorbidities significantly increase the risk of developing Alzheimer’s disease (AD) and other dementias later in life [95,96,97,98]. Identifying ways to reduce the cognitive burden of obesity and protect against the development of dementias will be essential to public health, as trends in AD parallel those of obesity continue to rise.

Compensating for brain energy deficits is a core feature of metabolic strategies to delay the onset and slow the progression of a number of neurodegenerative diseases and brain disorders. Ketones have been suggested as therapeutic due to their ability to directly fuel the brain despite aging and disease-induced impairments in cerebral glucose metabolism [99,100] and are currently being explored in clinical settings (NCT04701957, NCT03935854, etc.). The data presented here suggest that ketones may also reduce the brain energy deficit by improving the mitochondrial fuel efficiency within the hippocampus, and represent a plausible strategy for protecting cognitive health in adulthood.

## 5. Conclusions

Ketogenic diets are metabolically and cognitively relevant in healthy adult mice and may be an effective strategy for protecting against cognitive decline associated with aging and disease.

## Figures and Tables

**Table 1:** CON and KETO macronutrient profile by kcal%.

	CON	KETO
Component	kcal%	kcal%
Fat	13.4	90
Protein	28.7	4.7
Carbohydrate	57.9	2
Ketone Ester	0	3.3

**Table 2:** Primary antibody information and dilutions.

Target	Dilution	Host	Company	ID
DRP1	1:1000	Rabbit	Novus Biologicals	NB110-55288
pDRP1	1:1000	Rabbit	Cell Signaling	3455
OPA1	1:1000	Rabbit	Novus Biologicals	NB110-55290
OXPHOS	1:5000	Mouse	Thermo	45-8099
β-actin	1:1000	Rabbit	Cell Signaling	13E5
β-actin	1:1000	Mouse	Cell Signaling	8H10D10

## Data Availability

Not applicable.
